# A Race Against Time: Early-Onset Differentiation Syndrome Following All-Trans-Retinoic-Acid (ATRA) Therapy in Acute Promyelocytic Leukemia (AML-M3)

**DOI:** 10.7759/cureus.50042

**Published:** 2023-12-06

**Authors:** Kamran Ahmad, Mahnosh Saleh, Yasir Ali, Amna Yaseen, Muhammad Ali, Musa Kakakhel, Muhammad Azeem Hayat

**Affiliations:** 1 Internal Medicine, Hayatabad Medical Complex Peshawar, Peshawar, PAK; 2 Internal Medicine, Ayub Teaching Hospital, Abbottabad, PAK; 3 Medicine, Hayatabad Medical Complex Peshawar, Peshawar, PAK; 4 Endocrinology, Diabetes and Metabolism, Hayatabad Medical Complex Peshawar, Peshawar, PAK; 5 Pharmacology, Peshawar Medical College, Peshawar, PAK

**Keywords:** treatment strategies, management of ds, complications of atra therapy, pml-rara fusion protein, adverse reaction to atra, prompt intervention, atra syndrome, all-trans retinoic acid (atra) therapy, acute promyelocytic leukemia (apl), differentiation syndrome

## Abstract

This study reports a case of differentiation syndrome, a rare complication of ATRA (all-trans-retinoic-acid) therapy, observed in a 20-year-old male with acute promyelocytic leukemia (APML). Following the initiation of ATRA therapy for APML, the patient presented with fever, bleeding gums, bloody stool, and mouth ulcers. After 36 hours, he developed respiratory distress, hypotension, tachycardia, and hypoxemia, leading to the diagnosis of differentiation syndrome. ATRA therapy was promptly discontinued, and the patient, exhibiting type 1 respiratory failure, necessitated intubation. The management included hydroxyurea, dexamethasone, vasopressors, intravenous fluids, and furosemide. After seven days, significant improvement was observed, underscoring the importance of recognizing and promptly addressing differentiation syndrome in APML patients undergoing ATRA therapy. This case emphasizes the necessity of ATRA discontinuation, coupled with the judicious use of steroids and hydroxyurea, in the effective management of differentiation syndrome.

## Introduction

The distinguishing feature of acute promyelocytic leukemia (APL) is the halt in the development of leukemic cells at the promyelocyte stage. In approximately 95% of cases, there is a specific balanced translocation between chromosomes 15 and 17 (t15;17)(q24;q21), resulting in the production of the PML-RARA fusion protein [1]. APL is a rapidly growing cancer in which an excess of myeloblasts are found in the bone marrow and blood. When patients with APL receive treatment using all-trans-retinoic acid (ATRA) and arsenic trioxide (ATO), it can lead to a condition known as APL differentiation syndrome (DS) (APL DS) [2]. Although most individuals tolerate ATRA well, there are occasionally serious side effects, with ATRA syndrome being the most significant. Frankel et al. were the first to describe this syndrome in 25% of newly diagnosed APL patients they treated with ATRA. Symptoms typically appeared 2 to 21 days following therapy initiation. They were commonly accompanied by an increased white blood cell (WBC) count, fever, weight gain, dyspnea, pleural effusion, and pulmonary infiltrates on a chest radiograph. In some cases, patients also experienced renal failure, hypotension, and pericardial effusion. Five out of nine patients in a study were transferred to an intensive care unit and required mechanical ventilation; unfortunately, three patients did not survive [3]. DS is associated with severe complications if not treated promptly. One of the most crucial aspects of treating DS is early detection and timely corticosteroid initiation [4,5]. Our research includes the signs, symptoms, and steps that can be followed for prompt diagnosis and treatment of DS.

## Case presentation

In this section, we present a comprehensive clinical overview of a 20-year-old male patient of Asian descent with no significant past medical history. The patient's initial presentation to our tertiary care hospital was characterized by a constellation of symptoms spanning three weeks, including pyrexia, musculoskeletal discomfort, hemorrhagic gingiva, melena, and oral ulcerations. Upon admission, notable laboratory findings included a WBC (white blood cell) count of 44,000/ml with 87% neutrophils and 13% lymphocytes (normal: 5,000-10,000/ml), a hemoglobin level of 6.6 g/L (normal: 13-17 g/L), and a platelet count of 40,900/mm³ (normal: 150,000-300,000/mm^3^). The patient's physical examination primarily revealed mild anemia, while other clinical parameters remained unremarkable.

Peripheral blood smears illustrated the presence of 21% blast cells and a conspicuous 63% promyelocyte population. A subsequent bone marrow examination, complemented by molecular analysis detecting the PML-RARA (promyelocytic leukemia/retinoic acid receptor alpha) fusion gene, and flow cytometry, led to the definitive diagnosis of APL. The bone marrow exhibited characteristics of hypercellularity, with 32% blast cells and a predominant 58% promyelocyte population. Flow cytometric analysis demonstrated pronounced expression of CD13, CD56, and CD117, consistent with APL.

Upon diagnosis confirmation, the patient's therapeutic regimen commenced with ATRA administration, concomitant with transfusions of packed red blood cells and platelets to address the severe anemia and thrombocytopenia. As the patient neared the point of discharge, three days post-admission and two days of ATRA therapy, a sudden clinical deterioration ensued. This deterioration was characterized by an abrupt onset of cough, dyspnea, hyperthermia (temperature of 105°F), and diaphoresis. Notably, hemodynamic instability was evident, with a recorded blood pressure of 60/40 mm Hg, a tachycardic pulse rate of 177 beats per minute, and peripheral oxygen saturation plummeting to 78% while breathing ambient air. Auscultation of the patient's lungs detected diffusely distributed coarse crackles.

In response to these critical developments, our initial diagnostic considerations encompassed disseminated intravascular coagulation (DIC), neutropenic sepsis with potential pulmonary involvement, and DS, an acknowledged complication of ATRA therapy in APL patients. Subsequently, a systematic battery of investigations was promptly initiated to elucidate the etiology of the patient's deteriorating clinical status, while concurrently eliminating potential confounding factors.

Moreover, due to concerns regarding DS, the ATRA administration was promptly suspended. This critical decision was complemented by the initiation of broad-spectrum antimicrobial therapy and the implementation of supportive measures. The ensuing sections will provide a detailed exposition of the investigational findings, ultimately leading to the conclusive diagnosis of DS, warranting the patient's transfer to the intensive care unit (ICU) for a more specialized and intensive therapeutic approach.

**Figure 1:**
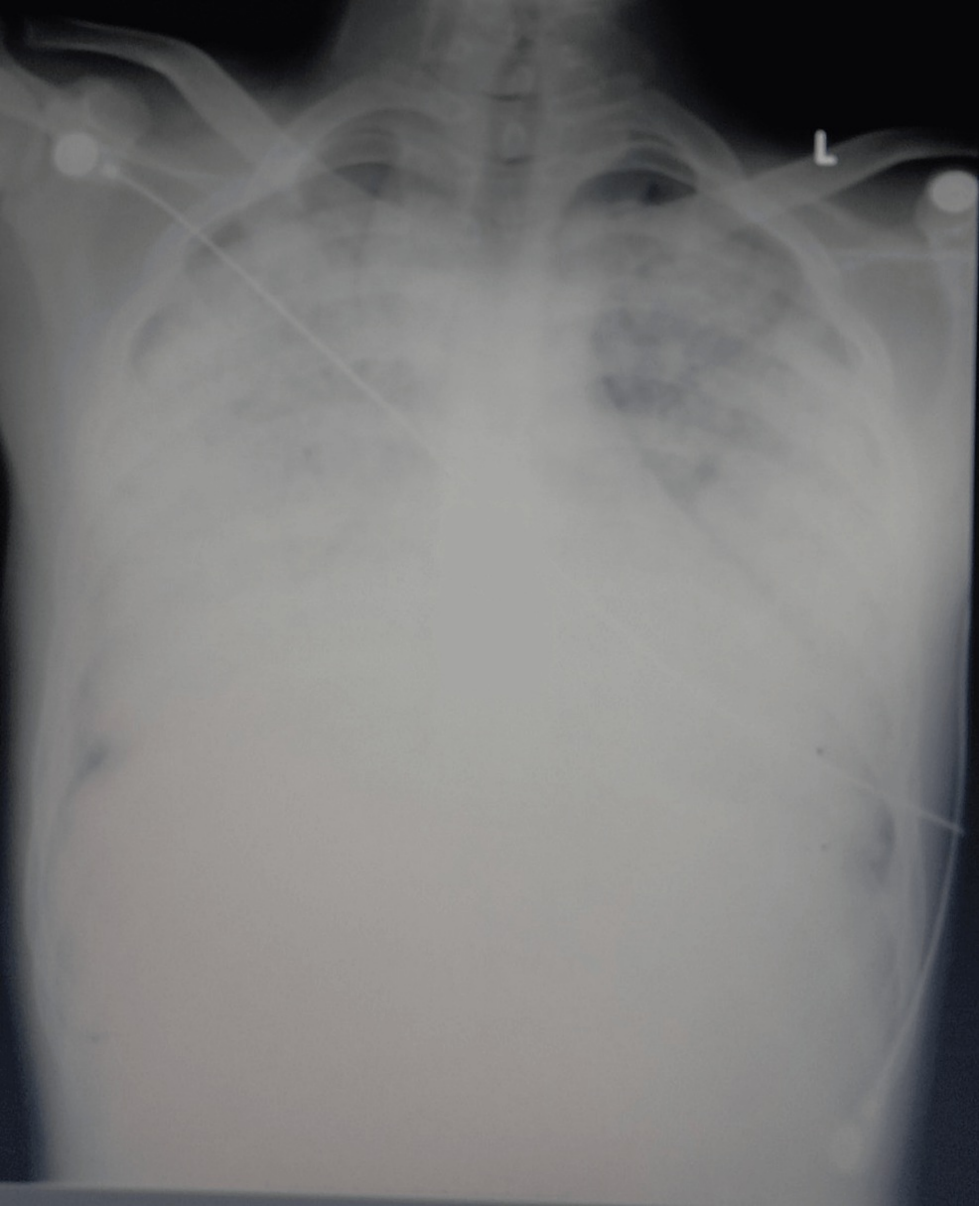
Chest X-ray showing bilateral infiltrates.

An echocardiogram (ECHO) was conducted, revealing a preserved ejection fraction and ruling out acute cardiac dysfunction. Notably, a chest radiograph (CXR) displayed bilateral diffuse infiltrations and haziness, as documented in Figure 1.

**Figure 2:**
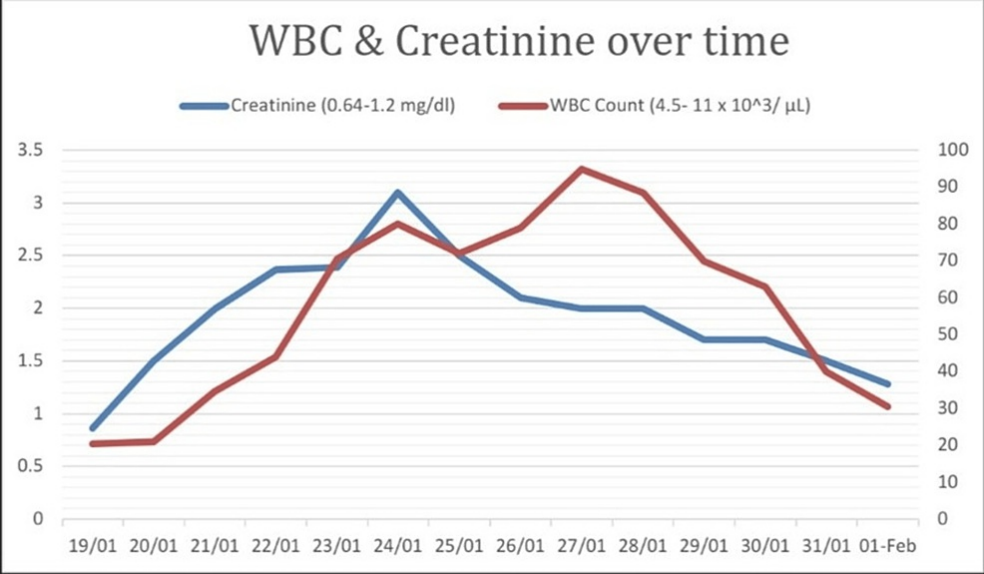
Trends of total WBC count and creatinine levels over the length of ICU stay.

The patient was subsequently admitted to the ICU, where the escalating severity of the clinical picture necessitated endotracheal intubation. Despite the immediate intervention, the patient’s white blood cell counts and renal markers (creatinine, blood urea nitrogen) continued to rise over the next week, reaching a peak of 95,000 cells per microliter and 3.1 mg/dl, respectively, as shown in Table 1 and Figure 2. Subsequently, the therapeutic strategy was augmented to encompass hydroxyurea (500 mg twice daily) and dexamethasone (10 mg twice daily), with the dosage later increased to 10 mg four times daily. Additionally, vasopressor support, intravenous fluid administration, and diuretics (furosemide) were judiciously administered. Over the ensuing days, the patient demonstrated an initial response to therapy, as evidenced by the resolution of early DS manifestations.

**Table 1:** Laboratory values at admission, after ATRA administration, halting, and post-hydroxyurea administration. BUN: blood urea nitrogen, TLC: total leucocyte count, ATRA: all-trans-retinoic-acid.

Date	Creatinine (0.64–1.2 mg/dl)	BUN (18–45 mg/dl)	TLC (4.5– 11 × 10^3^/μL)	Platelets (150–450 × 10^3^/μL)
January 19, 2023 (admission, ATRA started)	0.86	65.2	20.4	40.9
January 20, 2023	1.5	76	21	38
January 21, 2023 (ATRA stopped, Pt. shifted to ICU)	2	88	34.7	32
January 22, 2023	2.37	89	44	16.6
January 23, 2023 (platelet transfusion)	2.39	94	70.67	32
January 24, 2023	3.1	90	80	52.5
January 25, 2023	2.5	86	72	90
January 26, 2023 (extubated, clinically stable)	2.1	84	79	59
January 27, 2023 (hydroxyurea 500 mg BD initiated + increased steroid dose – oncology consultation)	2	80	95	41
January 28, 2023	2	75	88.5	76
January 29, 2023	1.7	68	70	70
January 30, 2023	1.7	64	63	74
January 31, 2023	1.5	60	40	78
February 1, 2023 (discharge ready to oncology team to restart ATRA)	1.28	63	30.4	80

**Figure 3:**
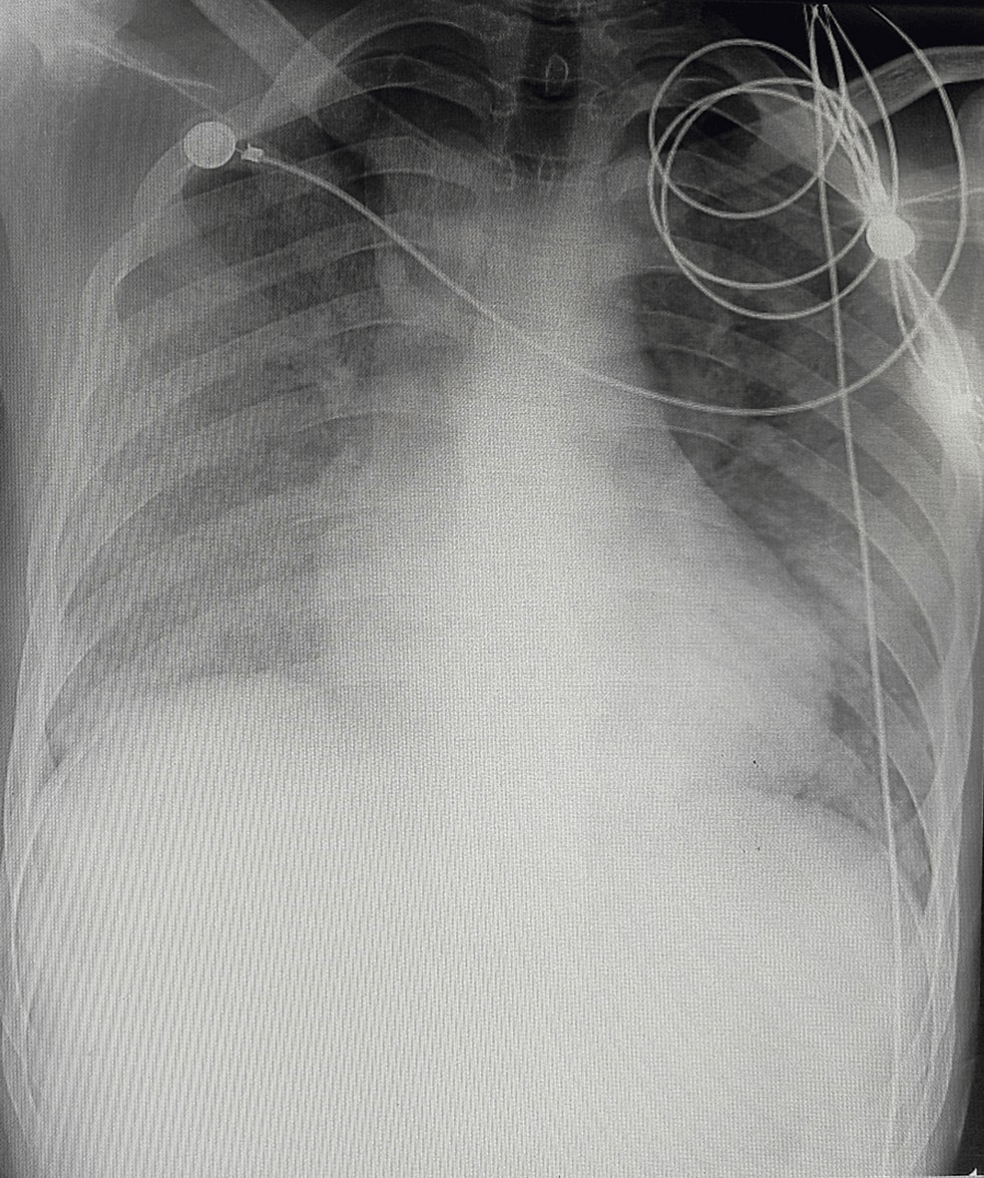
Chest X-ray showing improvement after ICU management.

Following his encouraging development, the patient was successfully extubated on the seventh day post-admission, with concomitant ATRA suspension. He was kept under observation for a period of five more days post-extubation to monitor his response to hydroxyurea and the increased steroid dose. An eleven-day stay in the ICU was marked by progressive clinical improvement, culminating in the final chest radiograph (Figure 3) documenting the substantial resolution of previously observed pulmonary infiltrates, and significant improvement in blood and renal markers. He was then shifted back to our ward and was subsequently discharged under the care of the oncology team, who restarted him on ATRA along with a retinoid.

## Discussion

When patients with APL receive induction therapy with ATRA and ATO, they may develop DS, also known as retinoic acid syndrome [6]. The underlying causes of DS in APL are complex and not fully understood. It is believed that ATRA has its effects by differentiating blast cells, leading to the production of various cytokines and altering the adhesive properties of these cells. This process results in the release of pro-inflammatory cytokines, such as interleukin 1 beta (IL1B), IL6, IL8, and tumor necrosis factor-alpha (TNF), leading to a systemic inflammatory response syndrome [2]. The published incidence of this syndrome, which is not fully understood, ranges from 2% to 27% [7]. The mortality rate is 1% when the patient is started on steroids in the early stages, compared to a 9% mortality rate without steroids [2]. In our case, when differentials were excluded along with other medications, he was started on steroids, the dose of which was later increased and played a role in his recovery. It is recommended to start the patient on a cytoreductive agent and, if there is no response within the first 24 hours, to increase the dose of steroids [2]. Early severe differentiation syndrome (which occurs during the first week of initiation of ATRA) is a rare event with a high death rate [7]. It is not advised to discontinue ATRA/ATO, but if there is severe APL DS, significant organ dysfunction, the need for intensive care unit care, or no improvement with steroids, discontinuation may be considered [2].

## Conclusions

In conclusion, this case study highlights the swift progression and life-threatening complexities of DS in APL patients undergoing ATRA therapy. The effective response, involving the temporary suspension of ATRA, initiation of broad-spectrum antibiotics, and implementation of high-intensity care, underscores the importance of prompt and adaptive strategies in managing acute DS manifestations.

Furthermore, the multidisciplinary collaboration among specialties, from hematology to ICU to radiology, played a crucial role in unraveling the clinical intricacies. This report significantly contributes to our understanding of DS, emphasizing the need for immediate action and reinforcing the imperative for clinicians to remain agile and vigilant in navigating the dynamic landscape of patient care.

## References

[1] Jimenez JJ, Chale RS, Abad AC, Schally AV (2020). Acute promyelocytic leukemia (APL): a review of the literature. Oncotarget.

[2] Stahl M, Tallman MS (2019). Differentiation syndrome in acute promyelocytic leukaemia. Br J Haematol.

[3] De Botton S, Dombret H, Sanz M (1998). Incidence, clinical features, and outcome of all trans-retinoic acid syndrome in 413 cases of newly diagnosed acute promyelocytic leukemia. Blood.

[4] Fidler KM (2020). Differentiation syndrome in patients with acute promyelocytic leukemia: what nurses need to know. Pediatr Nurs.

[5] Sultana J, Dutta J, Mustarin S, Dey P, Roy A, Mamoon MY (2022). Role of prophylactic steroids in differentiation syndrome. Cureus.

[6] Montesinos P, Sanz MA (2011). The differentiation syndrome in patients with acute promyelocytic leukemia: experience of the pethema group and review of the literature. Mediterr J Hematol Infect Dis.

[7] Montesinos P, Bergua JM, Vellenga E (2009). Differentiation syndrome in patients with acute promyelocytic leukemia treated with all-trans retinoic acid and anthracycline chemotherapy: characteristics, outcome, and prognostic factors. Blood.

